# Metabolism the Difficile Way: The Key to the Success of the Pathogen *Clostridioides difficile*

**DOI:** 10.3389/fmicb.2019.00219

**Published:** 2019-02-15

**Authors:** Meina Neumann-Schaal, Dieter Jahn, Kerstin Schmidt-Hohagen

**Affiliations:** ^1^Leibniz Institute DSMZ, German Collection of Microorganisms and Cell Cultures, Braunschweig, Germany; ^2^Integrated Centre of Systems Biology (BRICS), Braunschweig University of Technology, Braunschweig, Germany; ^3^Institute of Microbiology, Braunschweig University of Technology, Braunschweig, Germany; ^4^Department of Bioinformatics and Biochemistry, Braunschweig University of Technology, Braunschweig, Germany

**Keywords:** *Clostridioides (Clostridium) difficile*, metabolism, fermentation, TCA cycle, Wood-Ljungdahl pathway, Stickland reactions

## Abstract

Strains of *Clostridioides difficile* cause detrimental diarrheas with thousands of deaths worldwide. The infection process by the Gram-positive, strictly anaerobic gut bacterium is directly related to its unique metabolism, using multiple Stickland-type amino acid fermentation reactions coupled to Rnf complex-mediated sodium/proton gradient formation for ATP generation. Major pathways utilize phenylalanine, leucine, glycine and proline with the formation of 3-phenylproprionate, isocaproate, butyrate, 5-methylcaproate, valerate and 5-aminovalerate. In parallel a versatile sugar catabolism including pyruvate formate-lyase as a central enzyme and an incomplete tricarboxylic acid cycle to prevent unnecessary NADH formation completes the picture. However, a complex gene regulatory network that carefully mediates the continuous adaptation of this metabolism to changing environmental conditions is only partially elucidated. It involves the pleiotropic regulators CodY and SigH, the known carbon metabolism regulator CcpA, the proline regulator PrdR, the iron regulator Fur, the small regulatory RNA CsrA and potentially the NADH-responsive regulator Rex. Here, we describe the current knowledge of the metabolic principles of energy generation by *C. difficile* and the underlying gene regulatory scenarios.

## Clostridioides (Clostridium) Difficile

*Clostridioides (Clostridium) difficile* (Lawson et al., 2016) was discovered in 1935 as a commensal of healthy newborns (Hall and O’Toole, 1935). It was only in the late 1970s that *C. difficile* was recognized as a severe pathogen, responsible for antibiotic-related pseudomembranous colitis (Bartlett et al., 1978). In the last 20 years, an emerging number of nosocomial and community-acquired infections with symptoms ranging from mild diarrhea to pseudomembranous colitis and toxic megacolon was documented (Bartlett, 2006; Rupnik et al., 2009; Knight et al., 2015; Lessa et al., 2015). Major risk factors are antibiotic therapy, age and immunosuppression (Bignardi, 1998). The symptoms including severe intestinal damage are believed to be mainly caused by the two large clostridial toxins A (TcdA) and B (TcdB) and the binary toxin Cdt (Carter et al., 2010; Carman et al., 2011). *In vitro*, the toxins are predominantly produced in the stationary phase. Toxin production directly depends on the metabolic state of *C. difficile* (Karlsson et al., 2008; Neumann-Schaal et al., 2015; Hofmann et al., 2018). The number of genome-sequenced *C. difficile* strains with short-read sequences has increased up to 7000 (https://enterobase.warwick.ac.uk/, Alikhan et al., 2018), while the number of closed genomes remains at about 50 (e.g. Sebaihia et al., 2006; He et al., 2010; Riedel et al., 2017). Currently, several high quality annotations of the *C. difficile* genome are available, which serve as a solid basis for a systematic investigation of the transcriptome, proteome, metabolome, and for the construction of genome-scale metabolic models (Sebaihia et al., 2006; Monot et al., 2011; Larocque et al., 2014; Pettit et al., 2014; Dannheim et al., 2017a,b; Jenior et al., 2017; Kashaf et al., 2017).

## Fermentation Pathways

### Stickland Metabolism

**Figure 1:**
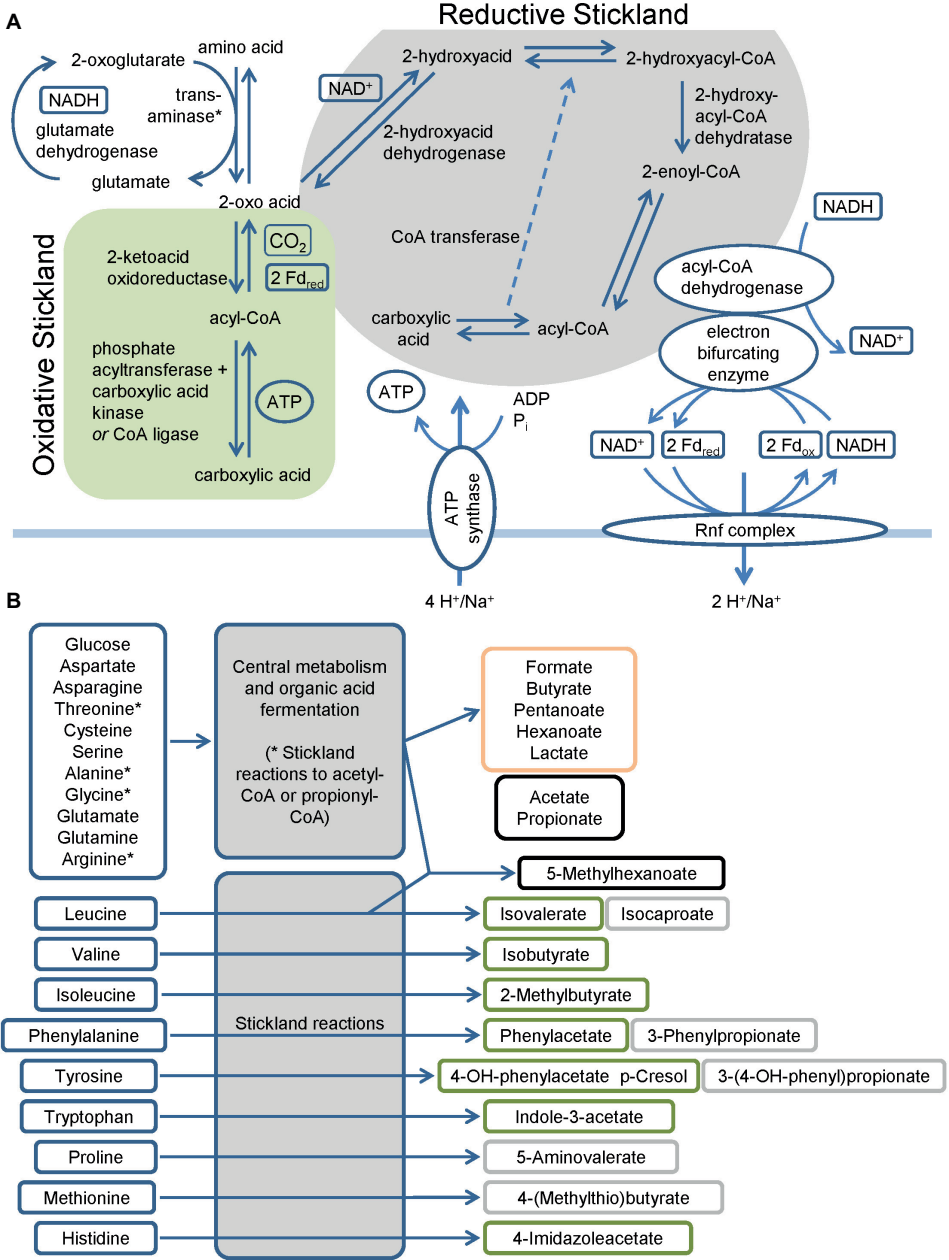
Overview of the fermentation metabolism in *Clostridioides difficile*. **(A)** Schematic overview of Stickland reactions showing the reaction steps of classical reductive and oxidative pathways and of the Rnf complex and the connection to the electron bifurcating enzymes. Products are shown at the end of the arrow and in boxes alongside the arrows, * Serine, threonine, methionine and cysteine are also subject to deamination by lyases. **(B)** Overview of amino acids and glucose as representative sugar and their fermentation products. The figure summarizes published fermentation products and substrates omitting alcohols and intermediates of the pathways for reasons of clarity. Corresponding alcohols are only minor products. (-OH: -hydroxy, Fd_ox_: Ferredoxin oxidized form, Fd_red_: Ferredoxin reduced form). Green: oxidative Stickland reactions and their products, gray: reductive Stickland reactions and their products, orange: central carbon metabolism-associated fermentation products, black: Stickland products (oxidative and/or reductive) and central carbon metabolism-associated fermentation products.

*C. difficile* harbors multiple pathways to utilize amino acids and sugars as energy sources (Mead, 1971; Elsden et al., 1976; Elsden and Hilton, 1979). The fermentation of amino acids via the so-called Stickland pathway occurs in three stages that ultimately couple the oxidation and reduction of amino acids to the formation of ATP (Stickland, 1934; Nisman, 1954). The first step is the transamination of an amino acid to its corresponding 2-oxo-acid (O’Neil and DeMoss, 1968; Barker, 1981) which yields NADH once coupled to the glutamate dehydrogenase reaction (Figure 1). In addition, serine, threonine, methionine, and cysteine are subject to deamination (Hofmeister et al., 1993; Morozova et al., 2013). The second part is either an oxidative or a reductive pathway. In the oxidative pathway, the formed 2-oxo acid gets oxidized by ferredoxin with the formation of a CoA thioester and the release of CO_2_ (Mai and Adams, 1994; Heider et al., 1996; Lin et al., 2015). The final steps of the pathway (encoded by *vorCBA, iorBA*) include the cleavage of the CoA thioester with subsequent ATP formation (Nisman, 1954; Valentine and Wolfe, 1960; Cary et al., 1988; Wiesenborn et al., 1989; Musfeldt and Schönheit, 2002). In the reductive pathway, the 2-oxo acid is reduced employing NADH with the formation of a 2-hydroxy acid (Hetzel et al., 2003; Martins et al., 2005; Kim et al., 2006). The dehydratation to enoyl-CoA (Dickert et al., 2002; Kim et al., 2004, 2005, 2008) in a CoA transferase reaction follows this (Kim et al., 2006). The reduction of the enoyl-CoA to acyl-CoA is catalyzed by an electron bifurcating acyl-CoA dehydrogenase (see below). The final step of the pathway is the transfer of the coenzyme A to the 2-hydroxy acid of the reductive path releasing the carboxylic acid (Kim et al., 2006) (Figure 1A). Genes of the reductive path are organized in the *hadAIBC-acdB-etfBA* operon with the exception of the *ldhA* gene which is localized upstream of the operon in the opposite direction. Interestingly, *C. difficile* revealed only a limited spectrum of amino acids utilized in the reductive pathway, while multiple amino acids can be used in the oxidative pathway (Figure 1B) (Elsden et al., 1976; Elsden and Hilton, 1978, 1979; Neumann-Schaal et al., 2015; Rees et al., 2016; Dannheim et al., 2017b; Riedel et al., 2017). Some amino acids are degraded by a modified Stickland pathway like the reduction of proline and glycine or the degradation of arginine via ornithine (Schmidt et al., 1952; Mitruka and Costilow, 1967; Hodgins and Abeles, 1969; Turner and Stadtman, 1973; Cone et al., 1976; Kabisch et al., 1999; Jackson et al., 2006; Fonknechten et al., 2009).

### Central Carbon Metabolism-Associated Fermentation

Besides the branched-chain and aromatic products of the Stickland reactions, *C. difficile* produces a number of straight-chain organic acids including acetate, lactate, propionate and butyrate (Neumann-Schaal et al., 2015; Rees et al., 2016; Dannheim et al., 2017b). Key metabolites for their formation are pyruvate and acetyl-CoA. With the exception of acetate, reducing equivalents are oxidized during the formation of the organic acids.

**Figure 2:**
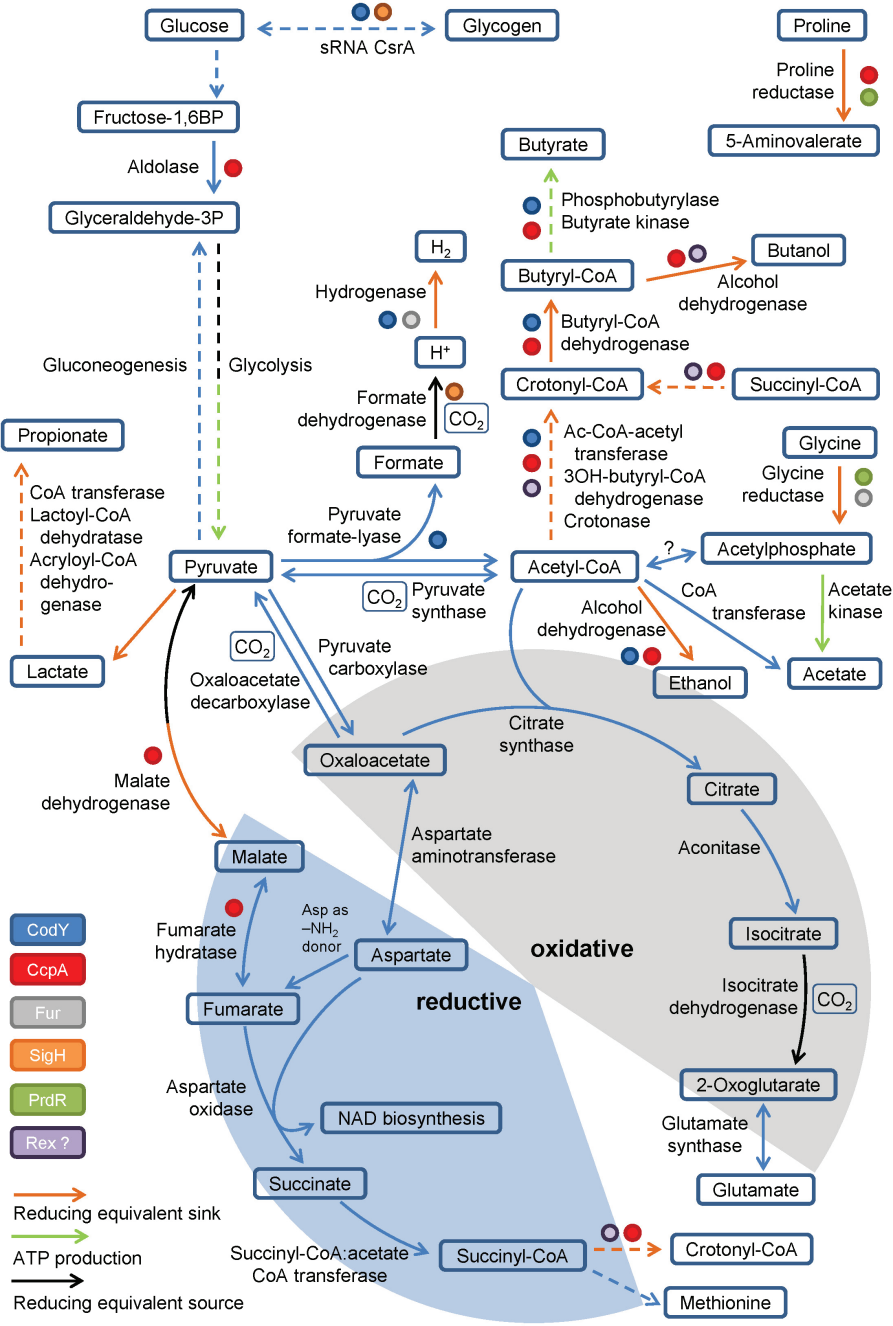
Glycolysis, gluconeogenesis, the fragmented TCA cycle, anaplerotic reactions and global regulators involved in metabolism. Schematic overview of the glycolysis, gluconeogenesis, the fragmented TCA cycle showing the oxidative and the reductive pathway, including anaplerotic reactions as well as global regulators controlling the central metabolism. Global regulators are marked by colored dots, ATP producing and reducing equivalent consuming/producing reactions are marked by colored arrows. (BP: bisphosphate, P: phosphate, Ac: Acetyl, -OH: -hydroxy). Dashed arrows represent multiple reactions.

Pyruvate, derived from carbohydrates and amino acids, is a key metabolite in both fermentation and the central carbon metabolism. It is fermented in two ways in *C. difficile*. It can be transformed to propionate via the reductive Stickland pathway as was observed in *Clostridium propionicum* (Hetzel et al., 2003; Schweiger und Buckel, 1984; Selmer et al., 2002). Or, it can be degraded to acetyl-CoA and produce butyrate via acetoacetyl-CoA and crotonyl-CoA (Figure 2). Clostridia typically use NADH to reduce acetoacetyl-CoA to 3-hydroxybutyryl-CoA (von Hugo et al., 1972; Sliwkowski and Hartmanis, 1984; Aboulnaga et al., 2013). The second reduction step of crotonyl-CoA to butyryl-CoA includes an electron bifurcating step (Aboulnaga et al., 2013) (see below). The enzymes of butyrate fermentation are organized in two operons (*bcd-etfBA-crt2-hbd-thlA* and *ptb1-buk*). Other products such as valerate and 5-methylhexanoate can be formed combining acetyl-CoA with propionyl-CoA or isovaleryl-CoA via the identical set of enzymes (Dannheim et al., 2017b). These reactions play a major role as a sink for reducing equivalents when favored substrates such as proline and leucine are not available (Neumann-Schaal et al., 2015).

### Electron Bifurcation and the Rnf Complex

Beside substrate-level phosphorylation, *C. difficile* couples several of the described fermentation pathways to the generation of a sodium/proton gradient using electron bifurcation in combination with the membrane spanning Rnf complex (Figure 1A). The Rnf complex was originally discovered in *Rhodobacter capsulatus* and catalyzes the reduction of NAD^+^ by ferredoxin (Schmehl et al., 1993; Biegel and Müller, 2010). Reduced ferredoxins can be produced through several ways. For instance, via ferredoxin-dependent oxidoreductases of the oxidative Stickland pathway or via dehydrogenases coupled to an electron bifurcation complex. Electron bifurcation couples the NADH-dependent reduction of a substrate (often CoA-derivatives) to the reduction of ferredoxin (Bertsch et al., 2013). This unique coupling is possible as the redox potential of enoyl-CoA (around 0 mV) is significantly higher than that of NAD^+^ (−280 mV) and ferredoxin (−500 mV) (Buckel and Thauer, 2013). Two electrons derived from NADH are distributed to two different electron acceptors, here an enoyl-CoA and ferredoxin. In *C. difficile,* electron bifurcating enzymes are found in several pathways including the reductive Stickland pathways (Figure 1A) and the butyrate/propionate fermentation pathways (butyryl-CoA und acryloyl-CoA dehydrogenases, Figure 2) (Hetzel et al., 2003; Aboulnaga et al., 2013; Bertsch et al., 2013). Finally, the free energy resulting from redox potential difference between ferredoxin (−500 mV) and NAD^+^ (−280 mV) is used to transport ions across the membrane (Buckel and Thauer, 2018). The nature of transported ions has not been studied in *C. difficile*. However, the transport of protons was observed for other clostridia (Biegel and Müller, 2010; Tremblay et al., 2012; Hess et al., 2013; Mock et al., 2015). Already in the 1980s, it was shown that also proline reduction is coupled to proton motive force generation (Lovitt et al., 1986), most likely via a direct interaction of the proline reductase with the Rnf complex. The generated ion gradient is used for either transport processes, motility, or for ATP generation via ATP synthase (Figure 1A).

What is the ATP recovery of the overall process? Usually organic acids are secreted in a protonated state. ATP synthase requires four ions for the generation of one molecule ATP (Dannheim et al., 2017b). The oxidative path reduces two molecules of NAD^+^ to NADH and phosphorylates 1.5 ADP. The reductive pathway regenerates one molecule NAD^+^ and produces 0.5 ATP. For leucine as substrate, the redox balance requires the reduction of two molecules leucine per one molecule oxidized leucine (Britz and Wilkinson, 1982; Kim et al., 2005). Overall, this leads to a production of 0.83 molecules ATP per molecule amino acid. Under the same conditions, the formation of acetate from acetyl-CoA yields 1.25 molecules ATP per molecule acetyl-CoA. The fermentation of two molecules acetyl-CoA to butyrate yields 1.75 molecules ATP and regenerates two reducing equivalents (Dannheim et al., 2017b).

## Central Carbon Metabolism

### Glycolysis and Gluconeogenesis and the Incomplete TCA Cycle

Pyruvate and acetyl-CoA are key metabolites used for a variety of different metabolic reactions in *C. difficile*. Pyruvate is produced via glycolysis and amino acid degradation (e.g. cysteine or alanine). Interestingly, cysteine and also pyruvate inhibit toxin production in *C. difficile* (Bouillaut et al., 2015; Dubois et al., 2016) emphasizing the tight connection of metabolism and pathogenicity. While glycolysis and gluconeogenesis follow classical pathways, acetyl-CoA can be produced via the Wood-Ljungdahl-pathway, via pyruvate synthase and via pyruvate formate-lyase.

For *Clostridium acetobutylicum* an incomplete TCA cycle was described (Amador-Noguez et al., 2011; Crown et al., 2011; Au et al., 2014). Based on genome annotation, *C. difficile* might also possess a truncated TCA cycle which is still sufficient for the production of biomass precursors and the degradation of nutrients (Dannheim et al., 2017b) (Figure 2). In *Clostridium kluyveri,* a citrate-(*Re*)-synthase is catalyzing the acetylation of oxaloacetate to form citrate replacing the common citrate-(*Si*)-synthase (Li et al., 2007). Citrate is further metabolized to 2-oxoglutarate (α-ketoglutarate), the precursor of the glutamate metabolism. Further oxidation of 2-oxoglutarate to succinyl-CoA is impaired as the 2-oxoglutarate synthase is missing. The reductive path is already interrupted at the level of oxaloacetate since a non-decarboxylating malate dehydrogenase is missing in *C. difficile* (Dannheim et al., 2017b). However, oxaloacetate is connected to fumarate via pyruvate and malate or aspartate. Aspartate serves as ammonium donor for arginine- and purine biosynthesis with the formation of fumarate. Fumarate can be degraded to pyruvate to refill the pyruvate pool or it can serve as electron acceptor for aspartate oxidase to produce iminosuccinate as a precursor of NAD biosynthesis (Senger and Papoutsakis, 2008; Dannheim et al., 2017b). The resulting succinate is a substrate of succinyl-CoA:acetate CoA transferase. The succinyl-CoA formed is used for methionine biosynthesis or is degraded to butyrate via crotonyl-CoA (Amador-Noguez et al., 2011; Crown et al., 2011; Au et al., 2014). In summary, the only reaction that contributes to the production of NADH in the truncated TCA cycle is the oxidation of isocitrate to 2-oxoglutarate (Figure 2). Other bacteria harboring a complete TCA cycle produce 3 NADH per acetyl-CoA, which is used for proton gradient formation and ATP generation. Since *C. difficile* is missing the classical electron transport chains, the TCA cycle is mainly used for the production and degradation of various metabolically important intermediates (Sebaihia et al., 2006).

### The Wood-Ljungdahl Pathway

Beside the already described reductive Stickland reactions and the butyrate fermentation, the Wood-Ljungdahl pathway also allows re-oxidation of NADH in *C. difficile*. In this pathway, which is also known as the reductive acetyl-CoA pathway, two molecules CO_2_ are used as terminal electron acceptors and reduced to acetate (Ragsdale, 1997). First CO_2_ gets reduced with NADPH to formate or directly into a formyl group by formate dehydrogenase. In a second step catalyzed by the carbon-monoxide dehydrogenase/acetyl-CoA synthase complex the formyl group is reduced to a methyl group and combined with CO and coenzyme A to acetyl-CoA (Ragsdale, 1997). Köpke et al. (2013) showed that the pathway is present in all 28 sequenced *C. difficile* strains available at that time. Moreover, they showed that the clinical isolate *C. difficile* 630 and closely related strains are capable of growing autotrophically on CO_2_ + H_2_. However, only slight growth was observed probably due to the lack of tryptophan biosynthesis (Sebaihia et al., 2006). Compared to true acetogens like *Clostridium ljungdahlii* (Köpke et al., 2010), *Moorella thermoacetica* (Pierce et al., 2008), and *Acetobacterium woodii* (Poehlein et al., 2012), *C. difficile* genomes only harbor an orphan acetate kinase gene. No obvious gene for a phosphotransacetylase was detected. However, this reaction might be catalyzed by the phosphotransbutyrylase (Köpke et al., 2013). In summary, fixation of the glycolysis-derived CO_2_ via the Wood-Ljungdahl pathway might be also a metabolic advantage for *C. difficile* in the human gut (Köpke et al., 2013).

### Pyruvate Utilization *via* Pyruvate Formate-lyase

*C. difficile* is utilizing pyruvate via the radical enzyme pyruvate formate-lyase, which forms the products acetyl-CoA and formate in the presence of coenzyme A (Figure 2). Pyruvate formate-lyase (PflD) requires an [4Fe-4S] cluster containing activating enzyme (PflC) for the formation of the catalytic glycyl radical (Crain and Broderick, 2014). The formate generated gets subsequently oxidized to CO_2_ and an electron by the formate dehydrogenase, a MoCo-containing selenoprotein (Pinske and Sawers, 2016). The electrons formed are transferred to a [NiFe] hydrogenase (Shafaat et al., 2013; Pinske and Sawers, 2016). Overall, the central metabolism in *C. difficile* mainly serves as an anabolic and catabolic hub and for CO_2_ fixation, avoiding the generation of NADH due to the lack of classical respiratory chains.

## Regulation of *C. Difficile* Energy Metabolism

Currently, only a partial view of the regulation of the *C. difficile* energy metabolism at the transcriptional and post-transcriptional level is available (Bouillaut et al., 2015). Clearly, the major regulator is the catabolite control regulator CcpA (Antunes et al., 2012). In *Bacillus subtilis* the LacI/GalR type regulator forms a complex with phosphorylated form of Hpr or Crh, which in turn is generated in the presence of high cellular glucose or fructose-1,6-bisphosphate concentrations (Fujita, 2009). In *C. difficile* about 140 genes are directly controlled by CcpA including genes of glycolysis, proline reduction, glycine reduction, butanol and butyrate formation (Figure 2; Antunes et al., 2012). In parallel, CcpA controls the expression of the toxin genes *tcdA* and *tcdB* in response to fructose-1,6-bisphosphate without phosphorylated Hpr, providing a strong link between metabolism and toxin production (Antunes et al., 2011). The proline-dependent regulator PrdR activates genes for proline reductase and represses the genes for glycine reductase (Figure 2; Bouillaut et al., 2013). The global regulator CodY provides a link to sporulation and another connection of the metabolism to toxin production (Dineen et al., 2007; Nawrocki et al., 2016; Ransom et al., 2018). In close cooperativity with the SinR and SinR’ proteins and the corresponding genes, CodY controls the toxin off state during the exponential growth phase (Girinathan et al., 2018; Ransom et al., 2018). Interestingly, culture heterogeneity caused by a bistable switch was observed (Ransom et al., 2018). CodY regulates also the energy metabolism via binding to promoters of genes involved in glycogen formation, the pyruvate formate-lyase path to hydrogen and butanol/butyrate generation (Figure 2; Dineen et al., 2010). The sigma factor SigH, controlling the genes of the glycogen metabolism and for formate dehydrogenase, represents another connection of the metabolism with sporulation (Saujet et al., 2011). The function of the NADH/NAD^+^-responsive regulator Rex in the fermentative metabolism of *C. acetobutylicum* was described before (Wietzke and Bahl, 2012). Participation in the regulation of butanol and butyrate formation in *C. difficile* was proposed (Figure 2; Bouillaut et al., 2015). The ferric uptake regulator Fur also directly influences glycine reduction and hydrogen formation from pyruvate in response to low iron condition (Figure 2; Ho and Ellermeier, 2015; Berges et al., 2018). Finally, the small regulatory RNA CsrA serves as carbon storage regulator influencing the genes of glycogen mobilization (Figure 2; Gu et al., 2018). Obviously, a complex regulatory network headed by the pleiotropic regulators CcpA and CodY co-regulates metabolism and toxin production. Similarly, CodY and SigH connect sporulation with the metabolism at the transcriptional level (Martin-Verstraete et al., 2016).

## Conclusion and Future Perspectives

In Western countries, hypertoxic *C. difficile* strains are causing several thousand deaths per year especially after antibiotic treatments. In this context, the mystery of the ecological success of this pathogenic bacterium is closely related to its unique and highly adaptive metabolism. Amino acids as building blocks of proteins are integral parts of our nutrition and thus available in access in our gut. Similarly, sugars from sugar polymers like starch constitute the carbohydrate part of our food. Both are the major energy sources of *C. difficile*. The versatile organism possesses multiple pathways for amino acid fermentation. However, normal substrate level phosphorylation suffers from very low ATP recoveries and the need to utilize parts of this ATP for ion gradient formation via a reverse ATPase reaction. Thus, smart *C. difficile* couples amino acid fermentation via electron bifurcation to membrane potential generating processes at the Rnf complex. Similarly, the central metabolism was modified to prevent unnecessary NADH generation, which usually has to be re-oxidized via energetically cost-intensive reactions. The organism uses an incomplete TCA cycle, generating one instead of three NADH. Furthermore, pyruvate formate-lyase instead of pyruvate dehydrogenase produces acetyl-CoA and formate, which gets transformed into protons by formate dehydrogenase and finally to hydrogen by a hydrogenase. Nevertheless, major metabolic fluxes have to be determined. Most likely, additional principles of energy generation will be uncovered. We are at the beginning of an exciting period of systems biology, allowing the integration of the different levels of cellular control represented by transcriptional control, RNA stability, translational control, metabolic control and coordinated degradation.

Currently, we know that this complex metabolism is controlled by a network of regulatory proteins, which directly connects it to toxin formation and sporulation. Major players are the catabolite regulator CcpA, the sporulation sigma factor SigH, the pleiotropic transcription factor CodY, the proline regulator PrdR, the iron responsive Fur and potentially the NADH/NAD^+^-ratio measuring Rex. Here, SigW and CodY are important players during the onset of sporulation. Similarly, CcpA and CodY regulate toxin gene transcription. A first small regulatory RNA (CsrA) was found involved in flagella formation, toxin production and host cell adherence. Most likely, this is only a small part of the yet unknown regulatory network underlying the efficient adaptation of the metabolism to changing environmental conditions. Novel regulatory principles including new regulators, novel small regulatory RNA and proteins, unknown changes in the protein–protein network with controlled proteolysis, direct metabolic regulation, control of RNA stability to name a few, have still to be elucidated.

## Author Contributions

MN-S, DJ, and KS-H wrote the manuscript.

### Conflict of Interest Statement

The authors declare that the research was conducted in the absence of any commercial or financial relationships that could be construed as a potential conflict of interest.
